# Expression of the Classical and Nonclassical HLA Molecules in Breast Cancer

**DOI:** 10.1155/2013/250435

**Published:** 2013-12-02

**Authors:** Gisela Bevilacqua Rolfsen Ferreira da Silva, Tarsia Giabardo Alves Silva, Roberta Aparecida Duarte, Nicolino Lia Neto, Hélio Humberto Angotti Carrara, Eduardo Antônio Donadi, Maria Alice Guimarães Gonçalves, Edson Garcia Soares, Christiane Pienna Soares

**Affiliations:** ^1^Department of Clinical Analysis, School of Pharmaceutical Sciences, UNESP, SP, Brazil; ^2^Department of Clinical Analysis, Laboratory of Cytology and Cell Biology, Faculty of Pharmaceutical Sciences, UNESP, Rua Expedicionários do Brasil 1621, 14801-902 Araraquara, SP, Brazil; ^3^Institute of Surgical Pathology and Cytopathology, Araraquara, SP, Brazil; ^4^Division of Clinical Immunology, Department of Medicine, Faculty of Medicine of Ribeirão Preto, University of São Paulo, SP, Brazil; ^5^Department of Pathology, Faculty of Medicine of Ribeirão Preto, University of São Paulo (USP), SP, Brazil

## Abstract

Considering that downregulation of HLA expression could represent a potential mechanism for breast carcinogenesis and metastasis, the aim of the present study was to use immunohistochemical methods to analyze the expression of HLA-Ia, HLA-DR, HLA-DQ, HLA-E, and HLA-G in invasive ductal carcinoma (IDC) of the breast and to relate this HLA profile to anatomopathological parameters. Fifty-two IDC from breast biopsies were stratified according to histological differentiation (well, moderately, and poorly differentiated) and to the presence of metastases in axillary lymph nodes. The expression of HLA molecules was assessed by immunohistochemistry, using a computer-assisted system. Overall, 31 (59.6%) out of the 52 IDC breast biopsies exhibited high expression of HLA-G, but only 14 (26.9%) showed high expression of HLA-E. A large number (41, 78.8%) of the biopsies showed low expression of HLA-Ia, while 45 (86.5%) showed high expression of HLA-DQ and 36 (69.2%) underexpressed HLA-DR. Moreover, 24 (41.2%) of 52 biopsies had both low HLA-Ia expression and high HLA-G expression, while 11 (21.2%) had low HLA-Ia expression and high HLA-E expression. These results suggest that, by different mechanisms, the downregulation of HLA-Ia, HLA-E, and HLA-DR and the upregulation of HLA-G and HLA-DQ are associated with immune response evasion and breast cancer aggressiveness.

## 1. Introduction

Breast cancer is the commonest neoplasm and the second cause of cancer death in women worldwide. It is estimated that in the world more than one million women are diagnosed with breast cancer every year, and more than 410,000 will die from the disease, representing approximately 14% of female cancer deaths [1].

Human leukocyte antigen (HLA) class I molecules have a central role in the cell-mediated immune system, especially as antigen-presenting molecules for cytotoxic T lymphocytes (CTLs), which can recognize tumor antigenic bound peptides, presented on the cell surface with HLA class I molecules, and kill the target cell [2, 3]. HLA-I expression seems to be lost or downregulated on the tumor cell surface and this might represent a mechanism for neoplastic cells to escape from being killed by CTLs, allowing tumor dissemination and metastasis [4].

HLA class II molecules (HLA-DR and HLA-DQ) are essential for peptide presentation to T-helper lymphocytes, and their expression may be responsible for triggering the immune response. Thus, the presence of these antigens may make the tumor more immunogenic, which could lead to a favorable prognosis. However, it has been proposed that HLA-DR molecules offer protection against NK cell cytotoxicity, as has been described for HLA class I antigens [5].

The class I human major histocompatibility complex (MHC) genes encode both the classical (extremely polymorphic) HLA-Ia (HLA-A, -B and -C) molecules and the nonclassical HLA-Ib (-E, -F and -G) molecules, characterized by low allelic polymorphism, limited tissue distribution, and the presence of membrane-bound and soluble isoforms. HLA-G and HLA-E expression at the tumor cell surface might allow it to escape T and natural killer (NK) cell immune surveillance. Surface HLA-E appears to confer protection from the NK cell-mediated lysis via the CD94/NKG2A receptor. Indeed, the role of HLA-G may be to interact with NK cell inhibitory receptors, such as ILT2 or ILT4 [6].

The purpose of the present study was to analyze the relation between HLA-Ia, HLA-Ib, and classical HLA-II (-DQ, -DR) expression in IDC of the breast and breast cancer aggressiveness and metastatic tumor behavior.

## 2. Material and Methods

### 2.1. Specimens

Tumor tissue specimens were taken at the Institute of Clinical Pathology (IPC) of Araraquara, state of São Paulo, Brazil. All tumors were classified as invasive ductal carcinomas (IDC) of the breast. Fifty-two IDC of the breast biopsies were analyzed by hematoxylin/eosin staining methods and stratified according to histological differentiation (well, moderately, and poorly differentiated) and the presence of metastasis in axillary lymph nodes. Tumors were measured and assigned to 3 size classes. Sections of 5 mm were cut and placed on organosilane pretreated slides.

### 2.2. Immunohistochemical Staining: HLA-I, HLA-DR, HLA-DQ, HLA-G, and HLA-E

A total of 52 formalin-fixed and paraffin-embedded biopsies of IDC of the breast were collected from 52 patients and stratified according to lesion grade. In the case of breast carcinoma with metastasis (16 patients), one biopsy of the respective axillary lymph node was also analyzed. Immunohistochemical tests with the streptavidin-biotin system (EP-USA/500, Signet, USA) were carried out, to detect the HLA-Ia, HLA-DQ, HLA-DR, HLA-G, and HLA-E antigens. Tissue specimens were dewaxed in xylene, rehydrated in graded alcohol, and rinsed in water. For antigen detection, the sections were immersed in 10 mM sodium citrate buffer, pH 6.0. Endogenous peroxidase was blocked by immersion in a hydrogen peroxide bath in absolute methanol (15 minutes each change) and nonspecific binding was performed with 3% low-fat dried milk diluted 1 : 100 in phosphate-buffered saline (PBS). Slides were incubated with the primary monoclonal antibodies (mAbs) for HLA-Ia (ab70328, diluted 1 : 50; ABCAM, Cambridge, England), HLA-DQ (ab55158, diluted 1 : 50; ABCAM, Cambridge, England), HLA-DR (ab175085, diluted 1 : 50; ABCAM, Cambridge, England), HLA-G (5A6G7, diluted 1 : 50; EXBIO, Prague, Czech Republic), and for HLA-E (MEM-E/02, diluted 1 : 50; EXBIO, Prague, Czech Republic) in a humidified chamber at 4°C overnight and then incubated with the streptavidin-peroxidase complex at 37°C for 30 min. The sections were then incubated in a solution containing 5 mg of diaminobenzidine (GIBBICO, Gaithersburg, Maryland, USA), dissolved in 5 mL of PBS, and 100 *μ*L of fresh peroxidase solution (450 *μ*L PBS and 50 *μ*L hydrogen peroxide) for 10 min, lightly counterstained with Carrazzi's hematoxylin without acid for 60 sec, exhaustively washed with tap water, air-dried, and mounted with Permount Mounting Medium (MERCK; Darmstadt, Germany).

**Figure 1:**
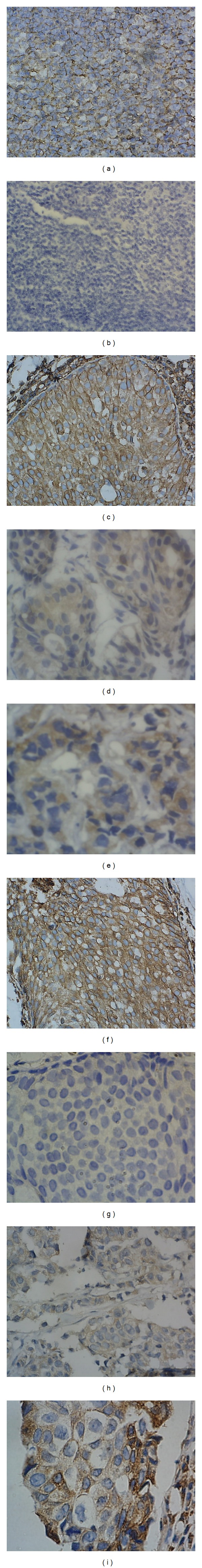
Immunoperoxidase labeling of HLA-Ia, HLA-II, HLA-G, and HLA-E in cytoplasmatic membranes of IDC breast tumor cells and controls. (a) Positive control for HLA-I, HLA-DR, and HLA-DQ expression in human tonsil slides, showing immunolabeling in >10%. (b) Negative control, human tonsil slides without primary antibodies, showing absence of immunolabeling. (c) HLA-I expression in IDC classified as positive, showing brown immunolabeling in >10%. (d) HLA-DQ expression in IDC classified as positive, showing intense brown immunolabeling in >10%. (e) HLA-DR expression in IDC classified as positive, showing intense brown immunolabeling in >10%. (f) Positive control for HLA-G and HLA-E expression in human trophoblast slide, showing immunolabeling in >25%. (g) Negative control, human trophoblast slide without primary antibodies, showing absence of immunolabeling. (h) HLA-G expression in IDC classified as positive, presenting labeling in >25%. (i) HLA-E expression in IDC classified as positive, showing labeling in >25%. Bar scale 250 *μ*m.

On each section, the mean number of positive-staining membranes was counted for both HLA-G and HLA-E immunostaining and classified as negative (absence of immunolabelling to 25% positivity) and positive (25% to 100% positivity). For HLA-Ia, HLA-DQ, and HLA-DR, immunostaining was classified as negative (absence of immunolabeling to 10% positivity) and positive (10% to 100% positivity) (Figure 1).

### 2.3. Computer-Assisted Analyses

Image analysis of tissues subjected to immunohistochemistry to determine the expression of HLA-Ia, HLA-DR, HLA-DQ, HLA-G, and HLA-E was performed in the image analyzer Image-Pro Plus (MD, USA). This equipment consists of a microscope (OLYMPUS BX50) coupled to a color camera (OLYMPUS DP10) and a dedicated computer containing the software, responsible for the mathematical determination of labeled cells. In each blade, an average of 10 fields was selected and digitized images were obtained in those fields in which it was established that the number of positive cells (marking in the cytoplasmic membrane) is 1000 cells/biopsy. Such quantification that could be interactively controlled by targeting through an RGB filter exists in software, allowing automatic retrieval of the number of positive cells. Thus, the average number labeled cells/biopsy of the 52 biopsies was determined and established the average percentage of positivity.

### 2.4. Controls

To validate the anti-HLA-Ia, anti-HLA-DQ, and anti-HLA-DR mAbs and the immunohistochemical method, we systematically analyzed a paraffin-embedded section of human tonsil (positive control). A negative control was prepared by omitting the primary antibody from the same tissue. To validate the anti-HLA-G and anti-HLA-E mAbs and the immunohistochemical method, a paraffin-embedded section of trophoblastic tissue from a third-trimester human placenta (positive control) was used. A negative control was prepared by omitting the primary antibody.

### 2.5. Statistical Analysis

Data were analyzed statistically with the program Instat Mac 2.01 (GraphPad, San Diego, CA, USA). To analyze the quantitative expression of HLA molecules, the nonparametric distribution Kruskal-Wallis test was used, followed by the Dunn Test for multiple comparison between pairs among the groups. This test was utilized since the data did not exhibit Gaussian distribution in the normality test. The *χ* ^2^ test and, when appropriate, Fisher's exact test were used to compare the quantitative immunohistochemistry results for HLA molecules, tumor size, histological grade, nuclear grade, and presence or otherwise of metastases in axillary lymph nodes. Differences were considered statistically significant when *P* < 0.05.

## 3. Results

Based on the tolerogenic functions of breast tumors, we investigated possible associations between the expression of HLA class Ia, class Ib (HLA-G and HLA-E), and class II (HLA-DQ and HLA-DR) in breast tissue (Figure 1).

**Figure 2:**
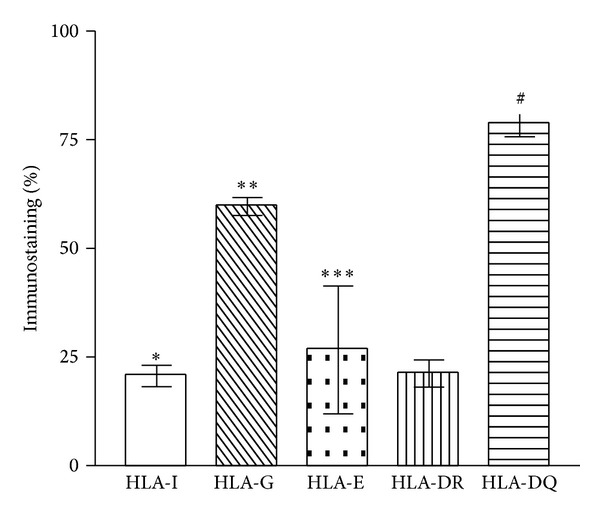
Quantitative expression of HLA class Ia (-A, -B, and -C), HLA class II (DR and DQ), and HLA class Ib (HLA-G and HLA-E) in invasive ductal carcinoma of the breast. Kruskal-Wallis, *P* < 0.001; post hoc Dunn's multiple comparison test, *HLA-I versus HLA-G and HLA-DQ (*P* < 0.001), and **HLA-G versus HLA-DQ, HLA-DR and HLA-E, (*P* < 0.01); ***HLA-E versus HLA-DQ (*P* < 0.001), ^#^HLA-DR versus HLA-DQ (*P* < 0.001).

The quantitative immunohistochemical assay results for HLA types, obtained by image analysis and expressed as mean proportion of positive-staining cells and standard deviation, are shown in Figure 2. A correlation was detected between low expression of HLA-Ia and high expression of HLA-G (*P* < 0.001), likewise between HLA-G high expression and HLA-E low expression (*P* < 0.01). No relation was found between HLA-Ia and HLA-E expression (*P* > 0.05). HLA-DR was significantly less expressed in IDC lesions than HLA-G and HLA-DQ molecules (*P* < 0.01) but at the same level as HLA class Ia and HLA-E. When HLA-DQ was compared with the other HLA molecules, it was found that its expression was raised in the invasive tumors a little more than the level of HLA-G. There was a significant difference between expression of HLA-DR and HLA-DQ (*P* < 0.001) that could indicate an association between high levels of HLA-DQ and low levels of HLA-DR expression.

**Figure 3:**
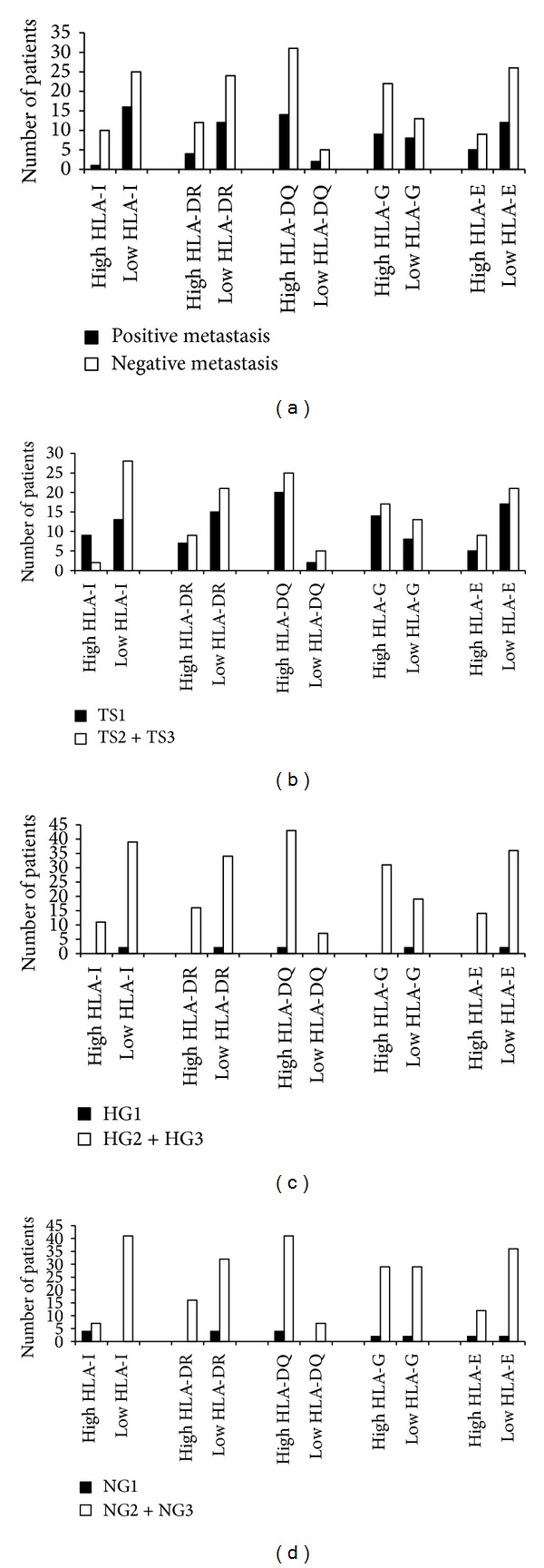
Quantitative immunohistochemical expression of HLA class Ia (-A, -B, and -C), HLA class II (DR and DQ), and HLA class Ib (HLA-G and HLA-E) in the 52 patients with invasive ductal carcinoma, associated with (a) metastases in axillary lymph nodes. *χ* ^2^ test: HLA class Ia (*P* = 0.2830; *P* > 0.05); HLA-II DR (*P* = 0.3010; *P* > 0.05) and HLA-II DQ (*P* = 0.6894; *P* > 0.05). HLA-G (*P* = 0.9512) and HLA-E (*P* = 0.3963; *P* > 0.05), (b) tumor size TS1 (<2 cm), TS2 (2 to 5 cm), TS3 (>5 cm). *χ* ^2^ test: HLA-Ia (*P* = 0.0683; *P* < 0.05); HLA-DR (*P* = 1.000; *P* > 0.05); HLA-DQ (*P* = 0.4420; *P* > 0.05); HLA-G (*P* = 0.5801) and HLA-E (*P* = 0.1826; *P* > 0.05), (c) histological grade (HG). *χ* ^2^ test: HLA-Ia (*P* = 0.0062; *P* < 0.05); HLA-DR and HLA-DQ (resp., *P* = 1.000; *P* > 0.05); HLA-G (*P* = 0.2695) and HLA-E (*P* = 0.7070; *P* > 0.05), and (d) nuclear grade (NG). *χ* ^2^ test: HLA-Ia (*P* = 0.0071; *P* < 0.05); HLA-DR (*P* = 0.5621), HLA-DQ (*P* = 1.000; *P* > 0.05). HLA-G (*P* = 0.4086) and HLA-E (*P* = 0.0661); *P* > 0.05.

The relationships between HLA-Ia, HLA-II, HLA-G, and HLA-E immunohistochemical expression and axillary lymph node metastasis are demonstrated in Figure 3(a). No association was found between HLA-Ia (*P* = 0.2830; *P* > 0.05), HLA-G high expression (*P* = 0.9512; *P* > 0.05), HLA-E (*P* = 0.3963; *P* > 0.05), HLA-DR (*P* = 0.3010; *P* > 0.05), and HLA-DQ (*P* = 0.6894; *P* > 0.05) and metastases in axillary lymph nodes of IDC patients.

The relation between the HLA immunohistochemical profile and tumor size is demonstrated in Figure 3(b). A correlation (*P* = 0.00683; *P* < 0.05) between tumor size and HLA-I expression was observed. Tumors larger than 2 cm showed low HLA-I expression in 53.8% of the cases; on the other hand, tumors classified as positive for HLA-I were observed in 3.8% of patients with a tumor <2 cm. Thus, the results suggest that bigger lesions, possibly with higher proliferative activity, showed lower HLA-I expression. High HLA-G expression was observed in 25.0% of lesions smaller than 2 cm and 30.7% of larger lesions. On the other hand, low expression was observed in 17.3% of patients with lesions under 2 cm and 26.9% with larger lesions. For the HLA-E molecules, high expression was observed in 9.6% of lesions smaller than 2 cm and 15.4% larger lesions. HLA-E low expression was observed in 32.7% of the smaller lesions and in 42.3% of the larger. However, no association was observed between HLA-G (*P* = 0.5801) and HLA-E (*P* = 0.1826) expression and tumor size. In the HLA class II results, no association was found between levels of expression (HLA-DR; *P* = 1.000 and HLA-DQ; *P* = 0.4420; *P* > 0.05) and tumor size, suggesting that there is no modification of the HLA class II levels with increasing tumor mass, an indirect indicator of tumor proliferation.

The results for HLA class I, class II, HLA-G and HLA-E, and histological grade (HG) are demonstrated in Figure 3(c). A significant correlation was observed between higher histological grades and HLA-I low expression (*P* = 0.0062; *P* < 0.05). Among patients with low expression of HLA-I, greater histological disorganization (HG2 + HG3) was observed in 75.0%, compared to 3.8% patients with more organized histological lesions (HG1). Thus, a relationship can be seen between HLA-I low expression and less histological differentiation. Higher HLA-G expression and greater histological disorganization (HG2 + HG3) were observed in 55.7%, while HLA-E high expression and lower histological differentiation (HG2 + HG3) were observed in 25.0% of IDC patients. On the other hand, low HLA-G and HLA-E expression was observed in the less differentiated lesions in, respectively, 40.4% and 71.2% of patients. However, no significant correlation was observed between lower histological differentiation and HLA-G regulation (*P* = 0.2695) or HLA-E downregulation (*P* = 0.7070). Also, no association was found between HLA class II (DR and DQ) expression (resp., *P* = 1.000; *P* > 0.05) and greater histological disorganization.

The HLA class I, class II (DR and DQ), HLA-G and HLA-E immunohistochemical results, and cellular atypia levels of IDC of the breast, classified as nuclear grade, are shown in Figure 3(d). Lower expression of HLA-I (*P* = 0.0071, *P* < 0.05) was observed in the higher nuclear atypia lesions (NG2 + NG3) in 78.8% patients. Only 21.1% of the patients did not show HLA-I downregulation, classified in this study as a positive response, and of these patients 7.7% presented lower nuclear atypia (NG1). Hence, it may be suggested that in the less differentiated tumors, with a greater number of cellular atypias, HLA-I downregulation could be expected. HLA-G and HLA-E high expression was observed in higher nuclear atypia lesions (NG2 + NG3) in, respectively, 53.8% and 21% patients. On the other hand, HLA-G and HLA-E low expression was seen in, respectively, 38.5% and 71.2% of the IDC analyzed. In the same way, no relation was found between lesser cellular differentiation and HLA-G downregulation (*P* = 0.4086) and HLA-E downregulation (*P* = 0.0661). No significant association between HLA class II DR (*P* = 0.5621) and DQ (*P* = 1.000) expression and the cellular atypia of IDC of the breast was observed, apparently indicating that the expression of neither molecule was altered by cellular atypia.

**Figure 4:**
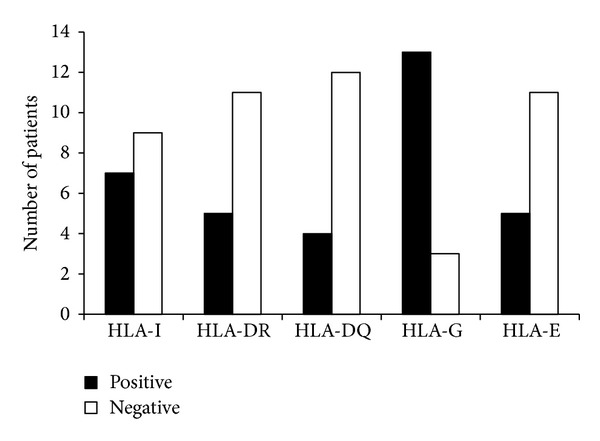
Immunohistochemical expression of HLA class Ia (-A, -B, and -C), HLA class II (DR and DQ), and HLA class Ib (HLA-G and HLA-E), in the tumor cells observed in the 16 lymph nodes of patients with metastases in axillary lymph nodes. Exact Fisher Test, *P* = 0.0091, *P* < 0.05.

To assess the similarities or differences in the expression of all HLA molecules investigated in the breast tumors and in their metastases in axillary lymph nodes, HLA class I, class II (DR and DQ), HLA-G, and HLA-E expression was assayed in the 16 lymph nodes of the patients that presented metastases (Figure 4). From these results, it can be seen that HLA class I, class II (DR and DQ) and HLA-E low expression and HLA-G (*P* = 0.0091, *P* < 0.05) high expression, in metastases in the lymph nodes, showed similar expression to that in the breast lesions. The results suggest a reduction in the expression of classical HLA molecules and an increase in HLA-G expression, related to the capacity for metastasis of this breast invasive tumor in axillary lymph nodes.

## 4. Discussion

The competent immune system has the capacity to recognize tumor cells and destroy them, preventing the seeding and growth of a series of tumors. Notwithstanding this, the same types of tumor cell may develop the ability to evade such immune control, by means of the loss or low expression of HLA class I molecules [4]. Decreased or absent HLA-I molecules expression has frequently been observed in a range of malignant neoplasms, unlike what occurs in normal breast tissue, in which the expression of HLA-Ia occurs [7–9]. However, the relationship between HLA-I expression and the process of carcinogenesis and metastatic progression of breast cancer has yet to be established with any certainty. The few studies carried out on this question have produced controversial results with regard to the deregulation of the expression of HLA-Ia and the potential for metastasis of the invasive ductal carcinoma of the breast [10]. It is well documented that tumor antigens are present in the lymphatic nodes nearest to the region of the neoplasm, either through the migration of malignant cells to the lymph nodes or by means of crossed presentation of molecules from the tumor on the antigen-presenting cells (APC) in the node [11]. This mechanism triggers the immune effector function but also generates some degree of tolerance to the development of the tumor. During tumor expansion, the presentation of tumor-specific antigen to the CD8+ T-cells by the APCs in the lymph nodes seems to lead to the proliferation of transitory effector cells, which is insufficient to trigger the functional response of the cytotoxic T-cells [12]. On the other hand, some studies demonstrate that changes in the expression of HLA class I and II molecules are related to early events in breast carcinogenesis and play an important part in the metastatic progression of breast IDC [5]. The deregulation of classical HLA-I is strongly associated with human breast cancer metastasis. The occurrence of metastasis in lymph nodes is generally taken to indicate a poor outcome for the breast cancer and heterogeneous HLA-I expression could be an additional sign of the preexistence and dissemination of the tumor [13]. The heterogeneous expression of class MHC molecules, frequently involving reduced levels or the complete absence of HLA-A, -B, and -C molecules, could reflect a major dissemination of tumor cells leading to metastasis of bone marrow and breast tumors [14]. In the present study, however, no significant association could be demonstrated between deregulation of HLA-I expression and metastasis in the axillary lymph nodes of patients suffering from IDC of the breast.

Few studies have assessed the relation between HLA expression and tumor size. In this study, an association was found between diminished expression of HLA-I and increase in tumor size. Larger breast tumors tend to be associated with worse patient outcomes and, when it is assessed, tumor size can be of use in predicting the evolution of the disease [15]. Although previous work has not revealed an association between immunohistochemical expression of the HLA antigens and tumor size [5, 10], the size of the neoplasm can demonstrate that a tumor has acquired the capacity to evade destruction by the immune system [11]. There is evidence that the modulation of the immune effector function is substantially weakened by the abnormal expression of growth factors or the loss of expression of antigens by the tumor [16]. Also, tumor cells are wrapped in stroma, whose extracellular matrix permits the anchoring of inflammatory cells, such as macrophages, granulocytes, and dendritic cells [11]. The immune cells found in the stroma can produce tumorigenic factors, as well as assisting in the evasion of the immune response by preventing the dendritic cells from maturing [17]. In the vicinity of a tumor, the T lymphocytes sometimes fail to migrate to the region because of the microenvironment created by the tumor itself, which strongly hinders T-cell migration [18]. In addition to losing the anchoring of T lymphocytes, the stroma adjacent to neoplastic tissue may help the tumor cells to multiply faster by promoting angiogenesis [12]. From the results reported here, it may be suggested that, in IDCs with a large tumor mass, there is a diminution of HLA-I expression, indicating the possible evasion of the immune response and thence a grave biological outcome.

Histological and nuclear grade, are histological parameters used to assess tumor differentiation, malignancy, prognosis and life-expectancy. Their correlation with a panel of clinical and pathological parameters may indicate the less differentiated character of breast tumors and the low expression of HLA-I has been closely associated with poorly differentiated tumors [14].

The histological aggressiveness and staging of lesions are vital data for the prognosis of breast cancer. Moreover, there is a consensus that nuclear grade, scored by a pathologist on the basis of the size and shape of the nucleus, can be used to predict the aggressiveness and potential for metastasis of malignant breast neoplasms [19]. In the present study, an association was found between the reduced immunohistochemical expression of HLA-I and the histological grade of the tumors [4, 5, 10]. Cell atypia is strongly indicative of the cell disarray found in malignant tumors, while abnormalities in the expression of classical HLA-I molecules have been described as a negative influence on the clinical course of the disease [14]. Thus, the present results demonstrate that larger lesions which show less histological differentiation exhibit anomalous HLA-I expression, possibly allowing the tumor cells evade the immune system and promoting the development of breast IDC.

In this study, quantitative analysis of the expression of classical and nonclassical HLA molecules revealed a negative correlation between HLA-I and HLA-G expression in IDC tissue, where HLA-I was found to be underexpressed and HLA-G overexpressed. An association between raised levels of HLA-G molecules and human carcinogenesis has already been noted and their expression by certain types of tumor is well documented, though some results are controversial [20–31]. It is well established that HLA-G molecules are expressed in trophoblast cells, thus warding off the host immune response so as to protect the fetus from attack by cytolytic cells [32]. Deregulation of HLA-G expression seems to be a common occurrence, associated with downregulation of HLA-I, and this may represent a pattern pointing to malignant transformation [33, 34]. A target showing resistance to the HLA-I mediated immune response in a tumor could exhibit a raised HLA-G response and it has been postulated that abnormal expression of nonclassical HLA molecules may be required to inhibit the signal to natural killer (NK) cells, making the neoplastic cells resist lysis and enabling them to avoid detection by the immune system [21]. Tumors can follow a number of paths to escape from NK cells [35] and thus both of these molecules could work together to keep the tumor cells alive within their microenvironment, favoring their proliferation and malignant progression.

An unexpected pattern of HLA-Ib expression was observed in this study, namely, low levels of HLA-E molecules in IDC lesions, combined with high levels of HLA-G. Generally, HLA-G is not expressed on normal cells (absence of malignance). Due to this factor, it was possible to check in our study that MEMG-9 Ab did stain in a significant number of tumor tissues. Normally, HLA-E surface expression is dependent on the availability of HLA class I signal sequence-derived peptides. Accordingly, HLA-E surface expression is generally considered to be coexpressed with classical HLA class I, which is expressed in the majority of healthy tissues [9, 33, 34].

Few lesions were found to express HLA-E and those that showed positive immunostaining did so at low intensity. The expression of HLA-E had been expected to rise alongside that of HLA-G, as had occurred previously in cells transformed by cytomegalovirus [36]. HLA-E is normally transcribed in various tissues, but is expressed weakly on the surface of the cells, where its stability depends on the coexpression of HLA-C, HLA-G, and HLA-A molecules [35]. The normal expression of class I HLA molecules allows the HLA-E complex to become stable and thus be expressed more strongly [35]. It was evident in our study that the fall in HLA-I expression, followed by overexpression of HLA-G, resulted in the inhibition of HLA-E expression. Earlier research has demonstrated that there is competition between HLA-E and class Ia molecules for the *β*_2_M (“light”) chains. Malignant transformation frequently changes the heavy : light chain ratio and could thus modify the level of HLA-E detected on the cell surface. In this situation, there is concomitantly a total loss, selective loss, or reduced expression of class I MHC molecules [37].

In this study, when the expression of HLA-G and HLA-E was compared with the presence of anatomopathological features, no association was found between deregulation of these HLAs and increase in tumor size, lower differentiation of the lesion, or axillary lymph node metastasis. It is possible that the higher expression of HLA-G and lower expression of HLA-E are not directly related to the aggressiveness or metastatic potential of the IDC. To our knowledge, no other study has investigated a possible correlation between these clinical features and the expression of the nonclassical HLA-G and HLA-E molecules.

Regarding the expression of class II molecules (HLA-DR and -DQ), the present results demonstrate an association between low HLA-DR and high HLA-DQ expression. An earlier study of HLA-DR, -DQ, and -DP expression in Langerhans cells in cervical neoplasia reported that HLA-DR expression was lower than that of HLA-DQ [38]. HLA-DR and -DQ molecules have different functions in the induction of the immune response to tumors. HLA-DR has a role in the proliferation of T-helper/inducer lymphocytes, while -DQ mainly modulates the cytotoxic, suppressor, or both activities of T lymphocytes. Contrary to the results of our study, the -DR and -DQ responses have most frequently been seen as interdependent, meaning that the function of HLA-DR is positively correlated with those of HLA-DQ and the levels of protein expression are similar [39]. In the papers published to date on HLA status in breast cancer, only HLA-DR was assayed (in class II molecules) and it was found to be expressed in low amounts, as was class I HLA [5, 40], corroborating the present results. Moreover, those authors pointed out that no association was observed between the anatomopathological features and HLA-DR expression, as in this study. The expression of HLA class II molecules (HLA-DR and -DQ) in the present study demonstrated an association between low HLA-DR and high HLA-DQ expression. Since HLA-DR has a role in the proliferation of T-helper/inducer lymphocytes and HLA-DQ mainly modulates the cytotoxic, suppressor, or both activities of T lymphocytes, it would be expected if there was, in addition to decreased expression of HLA-DR, decreased expression of HLA-DQ, due to its ability to stimulate cytotoxicity in T lymphocyte. Therefore, the low expression of HLA-Ia and HLA-DR may suggest a mechanism of escape for IDC of the breast from the T helper-mediated immune response. In addition, the rise in HLA-DQ levels could stimulate the suppressor T-cell response, assisting the tumor in evading immune recognition.

Currently it is established that Her-2 (human epidermal growth factor receptor-2), Estrogen Receptor (ER), and Progesterone Receptor (PR) are the most commonly used biomarkers and therapeutic targets in breast cancer patients. However, these biomarkers are not expressed in 17–30% of women with breast cancer which restricts the use of existing therapies [41]. The triple negative breast cancer phenotype, which means tumors that are negative for Her-2, ER, and PR, is even more aggressive and resistant [41, 42]. Current evidence has plainly established the heterogeneity of cancer and consequently demonstrates the role of other molecules involved in the behavior of breast cancer [43].

Assessment of the expression of HLA-Ia, Ib (-G and -E), and II (-DR and -DQ) molecules in axillary lymph node metastases has very rarely been carried out in previous studies of various kinds of tumor. In this study, a similar pattern of response was observed in the metastases and primary tumors, indicating that the low expression of HLA-Ia (-A, -B, and -C) and HLA-II (-DR) molecules and the high levels of HLA-G should be related to both primary tumor carcinogenesis and the metastatic capacity of breast IDC. In conclusion, the results reported here suggest that the metastatic capacity of this tumor is associated with deregulation of classical and nonclassical HLA molecules, which results in its evasion of the host immune system, enabling the tumor to form metastases in the axillary lymph nodes.
